# GET_PANGENES: calling pangenes from plant genome alignments confirms presence-absence variation

**DOI:** 10.1186/s13059-023-03071-z

**Published:** 2023-10-05

**Authors:** Bruno Contreras-Moreira, Shradha Saraf, Guy Naamati, Ana M. Casas, Sandeep S. Amberkar, Paul Flicek, Andrew R. Jones, Sarah Dyer

**Affiliations:** 1 European Molecular Biology Laboratory, European Bioinformatics Institute, Hinxton, UK; 2 Estación Experimental Aula Dei-CSIC, 50059 Zaragoza, Spain; 3 Institute of Systems, Molecular and Integrative Biology, University of Liverpool, Liverpool, UK

**Keywords:** Pangene, Plant genome, Gene annotation, Collinearity, Whole genome alignment, Presence-absence variation

## Abstract

Crop pangenomes made from individual cultivar assemblies promise easy access to conserved genes, but genome content variability and inconsistent identifiers hamper their exploration. To address this, we define pangenes, which summarize a species coding potential and link back to original annotations. The protocol get_pangenes performs whole genome alignments (WGA) to call syntenic gene models based on coordinate overlaps. A benchmark with small and large plant genomes shows that pangenes recapitulate phylogeny-based orthologies and produce complete soft-core gene sets. Moreover, WGAs support lift-over and help confirm gene presence-absence variation. Source code and documentation: https://github.com/Ensembl/plant-scripts.

**Supplementary Information:**

The online version contains supplementary material available at 10.1186/s13059-023-03071-z.

## Background

For a growing number of crops and plants, there are now multiple genome assemblies available in public repositories. These data are driving the analysis of the pangenome, the union of all known genomes of a species. For instance, recently published pangenome reports include staple crops wheat and barley [1, 2]. While these efforts have greatly advanced our understanding of the variability of genomes within species, they have also prompted a new class of problems, those related to annotating and naming genes across cultivars. Different strategies are possible. For instance, the barley pangenome consortium lifted-over gene models from three genotypes (Morex, Barke, HOR10350) to all other assemblies. This procedure biases the gene space to that of the reference cultivars. In contrast, in other species, fresh gene annotations have been produced for different individuals or sampled populations [3]. In this case, care should be taken to follow the same annotation protocols throughout to avoid inflating the number of population-specific genes [4] or to conserve gene identifiers.

In this context, it is useful to define a pangene, a gene model or allele found in some or all individuals of a species in a similar genomic location. A pangene should integrate additional naming schemes, e.g., so that a cluster of gene models can share a common identifier that links back to their original gene identifiers. A pangene set defines our current understanding of the total coding potential of a species and can assist in gene model curation, by providing a pool of possible gene models for assessment.

Pangenes can be produced by a variety of approaches, such as iterative mapping and assembly [5], local alignments of nucleotide sequences [6], molecular phylogenies of chromosome-sorted proteins [7], or as a secondary product of genome graphs [8–10]. Whatever the approach, a common use case for pangenes is to capture presence-absence variation (PAV) at the gene level. However, previous work has observed that absent gene models are rarely caused by complete sequence deletions; instead, they might not be expressed in certain conditions or the underlying genomic regions might contain genetic variants such that the criteria for calling a gene model are not satisfied. For instance, sequence variants in introns or splice sites can reduce evidence for a gene model [11]. Mapping transcript isoforms from orthologous loci is also a useful way to determine whether a gene model is intact [12].

Here we present an approach to identify and analyze pangenes in sets of plant genomes which can explicitly confirm or reject PAVs by lifting-over viable gene models on candidate genomic segments. This approach requires computing pairwise whole genome alignments (WGAs), which are then used to estimate gene model overlaps across individuals. Finally, pairs of overlapping genes are iteratively merged to produce pangene clusters. The algorithm produces pangene clusters that are not biased towards the reference annotation and that can optionally be used to refine individual gene model annotation with information from all cultivars. We benchmark this approach on diverse datasets that cover monocots and dicots, as well as small and large genomes.

## Results

### A protocol for calling pangenes based on whole genome alignments

**Fig. 1:**
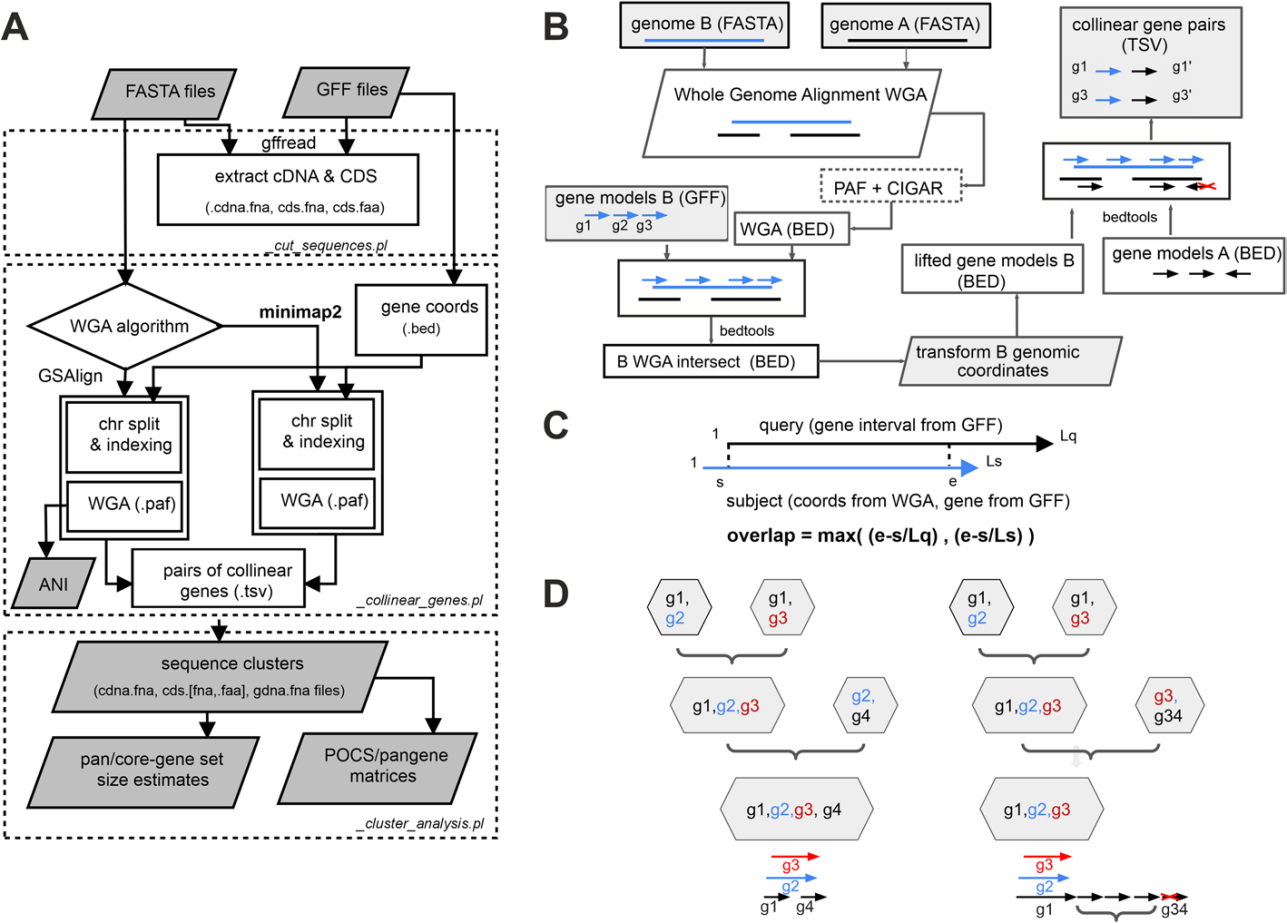
Features of get_pangenes.pl. **A** Flowchart of the main tasks and deliverables of script *get_pangenes.pl*: cutting cDNA and CDS sequences (top), calling collinear genes (middle, panels **B** and **C**) and clustering (bottom, panel **D**). By default, only cDNA and CDS sequences longer than 100 bp are considered. Whole genome alignments (WGA) can be computed with minimap2 (default) or GSAlign, and the input genomes can optionally be split in chromosomes or have their long geneless regions (> 1 Mbp) masked. Resulting gene clusters contain all isoforms and are post-processed to produce pangene and percentage of conserved sequences (POCS) matrices, as well as to estimate pan-, soft-core-, and core-genomes. GSAlign also produces average nucleotide identity (ANI) matrices. Several tasks can be fine-tuned by customizing an array of parameters, of which alignment coverage is perhaps the most important. **B** WGA of genomes A and B produces BED-like files that are intersected with gene models from B. Intersected coordinates are then used to transform B gene models to the genomic space of A. Finally, overlapping A gene models on the same strand are defined as collinear genes. **C** Feature overlap is computed from WGAs and gene coordinates from source GFF files. When checking the overlap of A and B gene models, strandedness is required. Overlaps can also be estimated between gene models annotated in one assembly and matched genomic segments from others. **D** Making greedy clusters by merging pairs of collinear genes. This algorithm has a key parameter, the maximum distance (in genes) among sequences of the same species that go in a cluster (default = 5). Its effect is illustrated on the right side, where gene g34 is left unclustered for having too many intervening genes

The first result from this work is the design of a protocol for calling pangenes in a series of related genomes. A pangene is defined as a gene model found within a homologous region in a set of genomes. In order to find pangenes, WGAs are computed, which in turn produce pairs of collinear genomic segments. Collinear evidence is stored in TSV files and can be produced by two WGA algorithms: minimap2 and GSAlign. The protocol is represented as a flowchart in Fig. 1A. As illustrated schematically in Fig. 1B, WGAs are used to project the coordinates of gene models across assemblies. By default, a pair of genes are said to be collinear when at least half the length of one matches the other in genomic space (Fig. 1C). Finally, clusters of genes (pangenes) emerge by merging pairs of collinear genes from different input taxa (Fig. 1D).

**Fig. 2:**
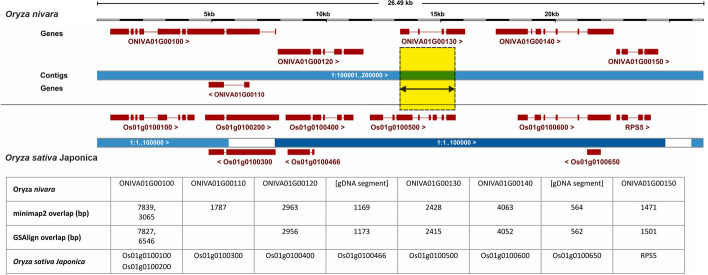
Aligned genomic region in chr1 of *Oryza nivara* (top) and *Oryza sativa* Japonica group cv. Nipponbare (bottom) as displayed in the Ensembl Plants browser. Genes on the forward strand ( >) are above contigs, whilst those in the negative strand ( <) are underneath. As a result of the genomic alignment, genes of *O. nivara* overlap with gene models from *O. sativa*. This evidence can be used to identify collinear genes that take equivalent positions in different genomes, as illustrated with gene models ONIVA01G00130 and Os01g0100500, which overlap over 2.4 kb (yellow rectangle). The example shows that overlapping gene models might share only some exons. The table below shows the collinear gene models identified based on minimap2 and GSAlign alignments, together with the corresponding overlapped base pairs

An example collinear region of *Oryza sativa* Japonica group (bottom) and *Oryza nivara* (dataset rice3) as displayed in the Ensembl Plants genome browser is shown in Fig. 2, together with a summary of the supporting WGA evidence. Besides five 1-to-1 collinear gene pairs, it can be seen that the gene ONIVA01G00100 was mapped to two consecutive *O. sativa* models (Os01g0100100, Os01g0100200) and that two *O. sativa* genes map to unannotated genome segments in *O. nivara*. In a nutshell, this figure shows that WGA evidence allows matching of long genes to split genes if they are collinear, as well as matching annotated gene models to homologous regions in other genomes, even if the genes in question failed to be annotated.

### Benchmark on several plant datasets

A systematic benchmark of *get_pangenes.pl* was performed with the four datasets in Table 1. In order to describe the performance several variables were collected. As computing WGAs is costly, particularly for large genomes, the maximum amount of RAM consumed by pairwise genome alignments was recorded. The size of the collinear fragments produced by WGA is captured in two variables, N50 and the percentage of fragments that contain blocks of 3 or more genes. Several further variables were also calculated to describe the clusters of collinear genes produced by the protocol: the total number of clusters, the number of pangenes present in all genotypes (core clusters), the pangenes present in 95% of genotypes (soft-core clusters), and the percentage of complete BUSCOs, which are universal single-copy orthologs. The results are summarized in Table 2 (minimap2) and Table 3 (GSAlign). Here the outcomes of both algorithms are compared. Although GSAlign consumes more RAM than minimap2 as genome size grows, this is not a fair comparison; in fact, while GSAlign was fed raw genome sequences, minimap2 only completed the WGAs for barley and wheat after masking long geneless genomic regions. The collinear segments aligned by minimap2 are longer and contain more genes than those produced by GSAlign. Additional file 1: Figs. S1 and S2 confirm that collinear gene models have large overlaps and that the genomic segments that contain them are generally syntenic along chromosomes. Additional file 1: Table S1 shows that the number of hits per gene is similar for both algorithms, with minimap2 failing to map more genes than GSAlign. Despite these differences, the numbers of pangenes clustered using WGA evidence from both algorithms are comparable: 86.3% of all clusters are identical (95.5% for core clusters, see Additional file 1: Fig. S3).

**Table 1:** Datasets used in this study. Geneset sources correspond to Ensembl Plants releases (EP) and the barley gene annotation from IPK [13]. Wheat genotypes are all *Triticum aestivum*. Barley genotypes are *Hordeum vulgare* landraces and cultivars, except B1K-04–12, which is *H. vulgare* subsp. *spontaneum*

Dataset	Species/genotype	INSDC accession	Geneset	Size (MB)	# Genes
ACK2	*Arabidopsis thaliana*	GCA_000001735.1	EP52	116	27,655
	*Arabidopsis lyrata*	GCA_000004255.1	EP52	200	32,667
rice3	*Oryza sativa* Nipponbare	GCA_001433935.1	EP54	363	37,960
	*Oryza sativa* indica 93–11	GCA_000004655.2	EP54	414	40,745
	*Oryza nivara* IRGC:100I897	GCA_000576065.1	EP54	327	36,313
chr1wheat10	Chinese Spring	GCA_900519105.1	EP52	1,736	18,017
	Arinalrfor	GCA_903993985.1	EP52	1,725	13,542
	Jagger	GCA_903993795.1	EP52	1,740	17,810
	Julius	GCA_903994195.1	EP52	1,729	17,669
	Lancer	GCA_903993975.1	EP52	1,721	17,951
	Landmark	GCA_903995565.1	EP52	1,738	17,792
	Mace	GCA_903994175.1	EP52	1,713	17,724
	Sy Mattis	GCA_903994185.1	EP52	1,718	17,941
	Norin61	GCA_904066035.1	EP52	1,726	18,091
	Stanley	GCA_903994155.1	EP52	1,737	17,767
barley20	MorexV2	LR722616-LR722623	IPK2020	4,210	46,294
	Akashinriki	ERS4201448	IPK2020	4,401	44,446
	B1K-04–12	ERS4201449	IPK2020	4,142	44,566
	Barke	ERS4201450	IPK2020	4,073	45,999
	Golden Promise	GCA_902500625.1	IPK2020	3,946	42,464
	HOR10350	ERS4201451	IPK2020	4,086	45,810
	HOR13821	ERS4201452	IPK2020	4,324	44,714
	HOR13942	ERS4201453	IPK2020	4,249	44,718
	HOR21599	ERS4201454	IPK2020	4,344	44,456
	HOR3081	ERS4201455	IPK2020	4,201	45,146
	HOR3365	ERS4201456	IPK2020	4,722	47,588
	HOR7552	ERS4201457	IPK2020	4,228	44,641
	HOR8148	ERS4201458	IPK2020	4,212	45,026
	HOR9043	ERS4201459	IPK2020	4,270	45,028
	Hockett	ERS4201460	IPK2020	4,201	46,450
	Igri	ERS4201461	IPK2020	4,202	45,213
	OUN333	ERS4201462	IPK2020	4,392	44,699
	RGT Planet	ERS4201463	IPK2020	4,213	45,413
	ZDM01467	ERS4201447	IPK2020	4,540	44,746
	ZDM02064	ERS4201446	IPK2020	4,153	45,050

**Table 2:** Summary of pangene analyses based on minimap2 whole genome alignments (WGA). N50 values, that describe the length of aligned genomic fragments, are shown as ranges of observed [min, max] values. The percentage of genes in blocks of 3 + contiguous genes is also shown as a range. Note that barley and wheat datasets require optional argument -H, which masks geneless regions longer than 1Mbp, where repeated sequences accumulate. Maximum RAM use was measured for pairwise WGA batch jobs

	Max RAM (GB)	WGA N50 (Kbp)	% Genes blocks3 +	Total clusters	(Soft) core clusters	% BUSCO complete
ACK2	4.5	6.1	34.0	38,785	20,647	94.1
rice3	1.4	[27.4, 29]	[75.1, 77.8]	61,913	19,170	85.2
chr1wheat10 (-H)	64.5	[80.8, 142.4]	[38.8, 53.8]	25,280	9937	
barley20 (-H)	46.3	[43.6, 75.7]	[25.4, 35.3]	165,544	23,811 (29, 226)	86.6 (95.3)

**Table 3:** Summary of pangene analyses based on GSAlign whole genome alignments (WGA). N50 values, that describe the length of aligned genomic fragments, are shown as ranges of observed [min, max] values. The percentage of genes in blocks of 3 + contiguous genes and the Average Nucleotide Identities (ANI) are also shown as ranges. Maximum RAM use was measured for pairwise WGA batch jobs

	Max RAM (GB)	WGA N50 (Kbp)	% Genes blocks3 +	Total clusters	(Soft) core clusters	% BUSCO complete	% ANI
ACK2	4.5	4.3	24.6	43,282	16,476	74.9	84.7
rice3	3.3	[15.2, 16.9]	[53.0, 57.9]	62,747	18,726	84.6	[96.4, 97.6]
chr1wheat10	83.4	[40.9, 72.1]	[20.4, 34.3]	30,005	7833		[98.9, 99.4]
barley20	113.1	[17.1, 34.3]	[10.8, 16.2]	168,880	15,567 (23,625)	61.6 (82.5)	[96.9, 99.3]

As an extra test, clusters of cDNA and CDS sequences resulting from the rice3 analysis were aligned locally to compute their sequence identity both at the nucleotide and protein level. Note that these clusters contain all isoforms annotated, so often there might be several sequences for the same gene. The results, plotted in Additional file 1: Fig. S4, yielded median sequence identities of 99.6% for nucleotides (cDNA and CDS). For protein sequences, the median values are 98.3% (GSAlign) and 98.1% (minimap2). This means that annotated sequences clustered together are nearly identical, although as seen in Additional file 1: Fig. S5, that does not guarantee that the same protein sequence is always encoded by clustered genes, as a result of divergent gene model annotation. Note that we also found some cases, 273/30,705 for minimap2 and 320/30,129 for GSAlign, where cDNA sequences of the same cluster could not be aligned. These occur when overlapping genes do not share exons. Nevertheless, these pangenes are not filtered out by default as such gene models could encode loss of function alleles that might be valuable to capture.

Tables 2 and 3 also contain a summary of BUSCO analysis. BUSCOs are sets of universal single-copy orthologs tailored to different taxa and are typically used to estimate the completeness of genome assemblies. In this context, BUSCOs provide a biologically meaningful metric based on expected gene content [14]. When we say an assembly is more “BUSCO complete” than others, it means it encodes more complete BUSCOs. The ACK2 dataset contains the two most divergent genomes, with 84.7% nucleotide identity (see Table 3). The core pangenes found across *A. thaliana* and *A. lyrata* contain a higher percentage of complete BUSCOs with minimap2 than with GSAlign (94.1% vs 74.9%). In the rice3 dataset, the nucleotide identity rises to 96% and the core pangene set contains ca. 85% complete BUSCOs with both WGA algorithms. As BUSCO analysis does not make sense for a single chromosome, the wheat dataset was left out. As for the barley20 dataset, the nucleotide identity is generally higher than that of rice3 and the benchmark produced core sets with close to 86.6% (minimap2) and 61.6% (GSAlign) complete BUSCOs. This number increased to 95.3% and 82.5% when all soft-core pangenes are considered, revealing a superior performance of minimap2 in this dataset. To put all these BUSCO scores in perspective, please see the scores of individual input genome annotations in Additional file 1: Table S2.

Finally, Table 3 also shows the average nucleotide identity values computed by the GSAlign algorithm for pairwise genome alignments. These values are useful to measure the divergence of the genomes being compared.

### Comparison to ancestral karyotype and Ensembl orthogroups

Additional analyses were carried out to gain insights into the performance of our protocol by comparing our results to data produced independently.

First, we estimated its recall on the ACK2 dataset, which represents the most difficult scenario tested due to having the lowest nucleotide identity. This experiment counted the number of collinear genes identified by minimap2 and GSAlign within 23 blocks of the Ancestral Crucifer Karyotype. The results, summarized in Additional file 1: Table S3, indicate that in these conditions, 65% (minimap2) and 52% (GSAlign) of the genes making up the blocks are called collinear.

Second, taking advantage of the fact that the genomes in datasets ACK2 and rice3 are included in Ensembl Plants, it was possible to compare the pangenes to precomputed Ensembl Compara orthogroups. In this comparison, clusters are said to match orthogroups when they include all the orthologues annotated in Ensembl; the results are shown in Table 4.

**Table 4:** Summary of pangene clusters obtained for datasets ACK2 and rice3 and the corresponding orthogroups in Ensembl Plants. Core clusters contain genes from all analyzed genomes; in rice, shell clusters contain genes from two species. BUSCO completeness percentages for core sets are shown in parentheses. Clusters with multiple copies have several genes from the same species. gDNA segments are shell clusters that bring together a gene model and a matching genomic segment from the underlying WGA. Column ‘match Compara’ shows the number of pangene clusters that contain the same genes as the corresponding Compara orthogroups. The last column shows the number of pangene clusters that contain sequences that share an InterPro domain (the number in square brackets is for core clusters only)

	Dataset	Core clusters [%BUSCO]	Multiple copies	Shell clusters	gDNA segments	Match Compara	Share InterPro domains
Compara orthogroups	ACK2	20,192 [90.6]	161				[18,259]
minimap2 clusters	ACK2	20,647 [94.1]	731			18,245	[18,792]
GSAlign clusters	ACK2	16,476 [74.9]	454			14,181	[14,817]
Compara orthogroups	rice3	13,020 [65.6]	219	6386			16,766 [11,571]
minimap2 clusters	rice3	22,880 [85.2]	3360	7825	6521	18,281	23,062 [19,239]
GSAlign clusters	rice3	20,399 [84.6]	2885	9730	6103	17,103	22,834 [17,135]

The analysis of the challenging ACK2 dataset shows that *get_pangenes.pl* recovers 90.3% (minimap2) and 70.2% (GSAlign) of core clusters found among Compara orthogroups, while producing more clusters with multiple copies. This means that ANI limits the recall of our protocol more severely for GSAlign than for minimap2. Nevertheless, 91% (18,792/20,647 minimap2) and 89.9% (14,817/16,476 GSAlign) of pangenes group together proteins that share InterPro domains, similar to Compara orthogroups (18,259/20,174; 90.5%). This indicates that pangene clusters are generally biologically relevant. Moreover, in the case of minimap2, the resulting core set is slightly more “BUSCO complete” than the Compara core.

The rice3 benchmark revealed that 79.9% (minimap2) and 83.8% (GSAlign) of pangene clusters match Compara orthogroups, with *get_pangenes.pl* calling over 7000 more core pangenes than Compara. As a quality check of these core pangenes, we counted how many encoded proteins share at least one InterPro domain. We found that 84.1% (19,239/22,880 minimap3) and 84% (17,135/20,399 GSAlign) of clusters are consistent in functional terms, compared to 89% (11,571/12,997 Compara). Although there seems to be a drop in the functional consistency of pangenes, this is compensated by their larger % BUSCO completeness (ca. 85% vs 65.6%).

Inspection of the examples on Additional file 1: Fig. S6 demonstrate that our protocol is able to cluster together overlapping gene models which might be split or incomplete in reference annotations [15, 16]. This would illustrate why pangene clusters are more likely to group together multiple sequences from the same species. Note that incomplete gene models, or clusters with sequences of contrasting length, would in turn explain why some clusters contain sequences that do not encode a common protein domain (see Additional file 1: Fig. S7). The table also shows that over 6000 shell pangene clusters are produced that pair an annotated gene model with an overlapping genomic segment (gDNA) from a different species. Additional file 1: Table S4 shows how core and shell clusters are represented as a BED-like pangene matrix produced by *get_pangenes.pl*.

### Confirming gene presence-absence variation

A use case of pangenome analysis of plants and other organisms is to find genes which might be present and functional only in some individuals or populations. These would be annotated by our pipeline as shell genes. As discussed in the previous section, the *get_pangenes.pl* protocol can produce gDNA sequence clusters that contain the genomic intervals of annotated gene models plus overlapping unannotated segments from other species. This feature takes advantage of pre-computed WGAs, which match genomic segments whether they harbor gene models or not. Such clusters can effectively be used to lift-over or project gene models from the species where they are annotated to other individuals. In particular, CDS or cDNA isoform sequences from annotated genes can be mapped to the matching genomic segments and the resulting alignment will directly confirm whether exon/intron boundaries and the embedded coding sequence are conserved. When conserved, the segment likely contains an overlooked gene model; when not, it probably contains a gene fragment or a pseudogene. A flowchart in Additional file 1: Fig. S7 summarizes how the script *check_evidence.pl* retrieves the WGA evidence supporting a pangene cluster, defines consensus and outlier isoform sequences, and then projects consensus CDS or cDNA sequences on candidate genomic segments with GMAP (see “Materials”). Note that, in addition to missing gene models, lifting-over can also merge split gene models or, conversely, divide gene models that might have been merged during gene annotation. Either way, when the projection succeeds and a complete open reading frame is aligned, a patch GFF file is created that conveys the genomic coordinates of the projected gene.

**Fig. 3:**
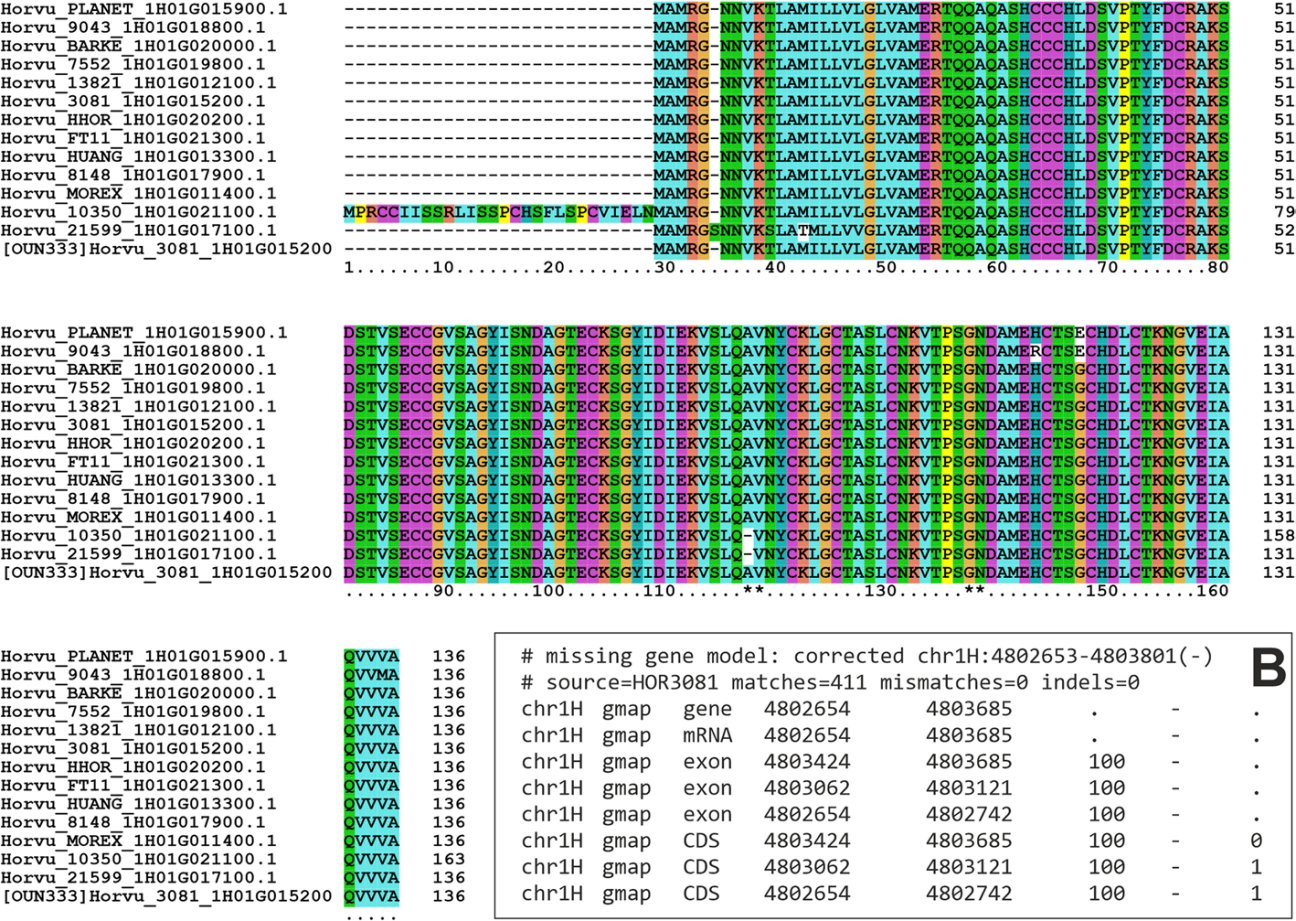
Multiple alignment of protein sequences encoded in barley pangene cluster Horvu_MOREX_1H01G011400, produced with Clustalx. This cluster contains isoforms from 13 gene models, but none from genotype OUN333. The last sequence is encoded by a CDS sequence lifted-over from cultivar HOR3081 on the genome of OUN3, spanning 3 exons (exon boundaries are marked with asterisks. B, Patch GFF file with the coordinates of the exons lifted-over from gene model Horvu_3081_1H01G015200. The underlying CDS nucleotide sequence was aligned with 411 matches, no indels and no mismatches with check_evidence.pl -f

Using shell CDS clusters of occupancy > 9 resulting from the minimap2 analysis of dataset barley20, we carried out a survey to see how often the different scenarios (missing, split, merged gene) occur in a real dataset. Out of 41,655 clusters, our approach detected 74 cases where a long model could be potentially corrected, 30 cases of incorrectly split genes, and 9839 potentially missing genes. We selected one candidate missing gene to illustrate the most common situation, pangene Horvu_MOREX_1H01G011400. In this example, the original pangene grouped together gene models from 13 barley genotypes, supported by the WGA evidence shown in Additional file 1: Table S5. When CDS nucleotide sequences from those 13 cultivars were aligned to candidate genomic segments of the remaining genotypes, a perfect match was found in the genome sequence of OUN333. The encoded lifted-over protein sequence is shown at the bottom of the multiple alignment in Fig. 3, being identical to others in the cluster. The resulting patch GFF file that would add this gene model to OUN333 is shown in Fig. 3B. This test case suggests the combination of WGA evidence and gene model projection could be a powerful way to refine gene annotation across individuals of the same species. Moreover, as shown in the example, lift-over alignments can be used to confirm or reject observed PAV. In this case, we can hypothesize this gene model is actually present in cultivar OUN333 and probably missing in the remaining genotypes. Additional data beyond sequence evidence would be required to fully characterize such genes, such as expression data and, ultimately, proteomics evidence.

### Curation of barley flowering genes

The protocol for pangene clustering was further tested with an increased set of barley assemblies and gene annotations. In this final experiment, the barley20 dataset plus the high-quality MorexV3 and BaRTv2 gene annotations were pooled and a subset of 26 genes known to regulate flowering and spike architecture extracted from the resulting clusters. The curated results are summarized on Table 5, where it can be seen that in all cases these genes were found in 19 or more barley annotations. The results in this gene survey were curated and we found some cases (HvFT3/Ppd-H2, HvLUX, HvGRP7b, HvLHY) where a gene was missing from a cluster in some cultivars but there was a candidate genomic region harboring part of that sequence. That would be the case for cultivars Igri and HOR3081 and locus HvFT3/Ppd-H2. As explained in the previous section, we lifted-over the collinear CDS nucleotide sequences from all the other cultivars and we obtained identical matches but only for exon 4 (see Additional file 1: Fig. S9), replicating previous observations that exons 1 to 3 of this gene have been deleted in some genotypes [17].

**Table 5:** Survey of flowering-related pangenes in a collection of 22 barley genotypes, including the barley20 dataset plus the MorexV3 and BaRTv2 high-quality annotations. Column “Occup” indicates how many annotations contain a gene model, with the number in brackets being the number of matched genomic segments in cases where a gene was absent in some cultivars. Column “Split” tells how many gene models are split in each pangene cluster with respect to the mode gene model. Column “Tand” says how many extra tandem gene models are in each cluster. Column ‘Inv’ states how many genes were found inverted in whole genome alignments

Locus	Gene identifier in MorexV3	Occup	Split	Tand	Inv
HvCO9	HORVU.MOREX.r3.1HG0058180	22			
HvFT3/Ppd-H2	HORVU.MOREX.r3.1HG0077240	19 [2]			1
HvELF3	HORVU.MOREX.r3.1HG0095050	22			8
HvPRR37/Ppd-H1	HORVU.MOREX.r3.2HG0107710	21			1
HvBM3	HORVU.MOREX.r3.2HG0127410	22			
HvBM8	HORVU.MOREX.r3.2HG0156870	22			
HvCEN	HORVU.MOREX.r3.2HG0166090	21			8
Vrs1	HORVU.MOREX.r3.2HG0184740	22			
HvGI	HORVU.MOREX.r3.3HG0238250	22			
HvFT2	HORVU.MOREX.r3.3HG0244930	22			
HvOS2	HORVU.MOREX.r3.3HG0311160	22	1		
HvLUX	HORVU.MOREX.r3.3HG0328340	19 [1]			4
HvGRP7a	HORVU.MOREX.r3.4HG0333810	22			1
PRR59	HORVU.MOREX.r3.4HG0350680	22	20		
HvFKF1	HORVU.MOREX.r3.4HG0369880	22			2
HvPRR73	HORVU.MOREX.r3.4HG0385940	22			
HvGRP7b	HORVU.MOREX.r3.5HG0421460	20 [1]		1	1
HvELF4-likeA	HORVU.MOREX.r3.5HG0478460	22			
HvPRR95	HORVU.MOREX.r3.5HG0498830	22			
HvZTL	HORVU.MOREX.r3.6HG0560010	22			
HvTOC1	HORVU.MOREX.r3.6HG0595250	21			1
HvCO2	HORVU.MOREX.r3.6HG0611620	22	1		
HvFT1	HORVU.MOREX.r3.7HG0653910	22			
HvCO1	HORVU.MOREX.r3.7HG0671540	22			1
HvLHY	HORVU.MOREX.r3.7HG0699010	20 [1]			
HvZTLa	HORVU.MOREX.r3.7HG0729460	22			2

Another interesting case was locus HvPRR37/Ppd-H1, which was found to be absent also in cultivar Igri, despite the gene being cloned in this cultivar with accession AY970701.1 [18]. As it turns out, the Igri genome assembly placed this gene model (Horvu_IGRI_Un01G026500) outside of the pseudo-chromosomes; instead, it is located in chrUn, and therefore, it cannot be collinear to the homologous genes in other cultivars when only homologous chromosomes are compared (option -s). This error can be avoided by not using option -s, which in practice means that chromosomes are compared all against all.

We also found several instances where genes were found in inverted genome regions, known to be valuable to reconstruct the history of crops [19]. In the case of HvCEN, shown in Additional file 1: Fig. S10, this observation matches previous reports [1], but we found other cases such as HvELF3 or HvLUX. These examples show the value of using whole genome alignments for the definition of pangenes, as the strand of genes conveys chromosomal and evolutionary information.

Among the curated genes, there’s also Vrs1, a homeodomain-leucine zipper homeobox gene known to control row number in barley spikes [20]. Some alleles of this locus encode proteins with frameshifts that change the phenotype and result in amino acid sequences that cannot be properly aligned. Additional file 1: Fig. S11 shows that the WGA-based strategy tested here was able to cluster together these alleles despite their low terminal protein identity.

**Fig. 4:**
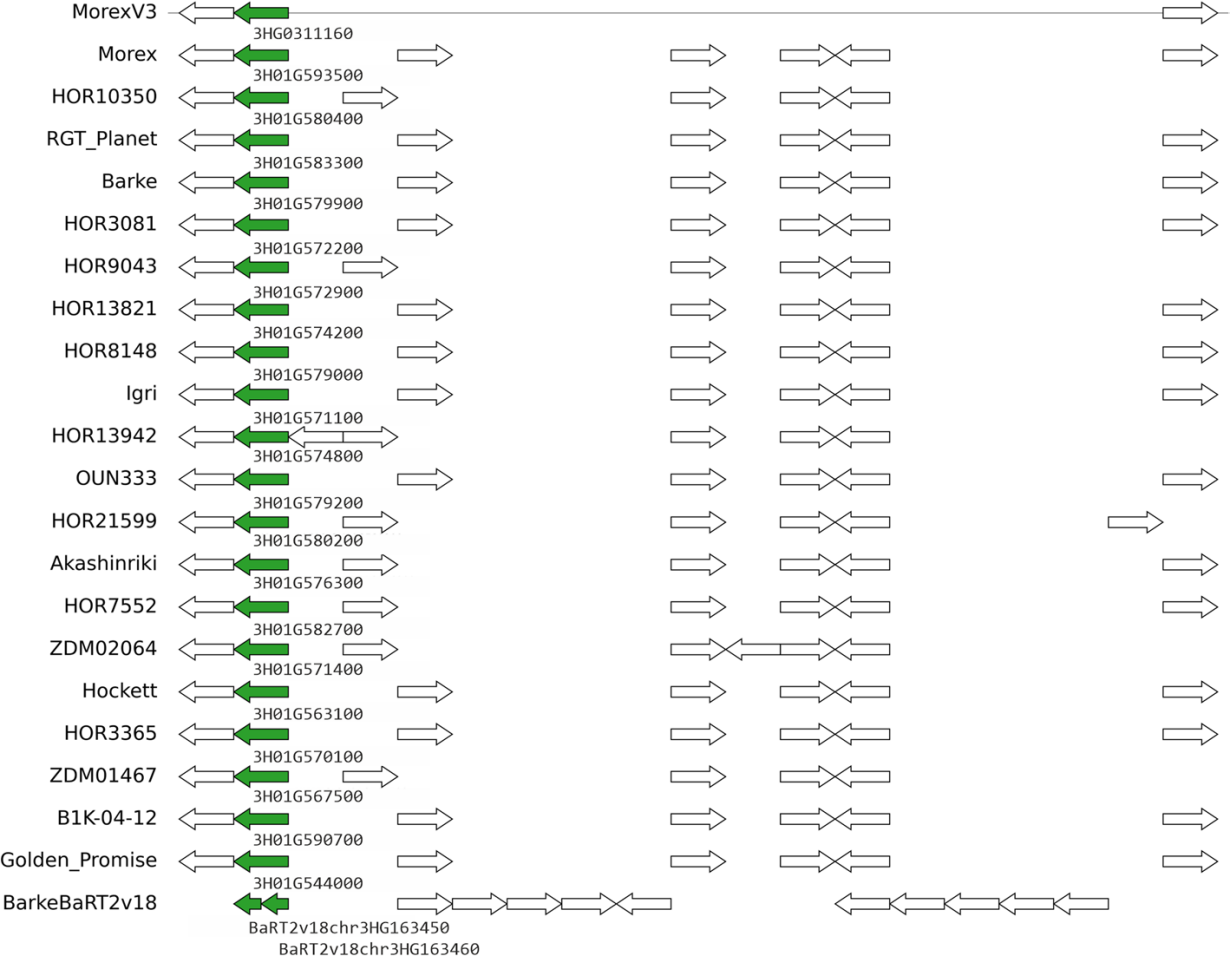
Genomic context of pangene cluster HORVU.MOREX.r3.3HG0311160 (green arrows), which corresponds to barley locus HvOS2. The genome fragment on top corresponds to reference genome MorexV3 and the tracks below show collinear genes found in other barley assemblies and annotation sets. In this example, the BarkeBaRT2v18 gene is split in two partial models. Note that white gene models might not be collinear as they could be encoded in a different genome fragment. Figure generated with script check_evidence.pl and pyGenomeViz (https://github.com/moshi4/pyGenomeViz)

Finally, in this set of genes used by barley breeders, we also observed instances of gene models found to be split in some annotations or genes with extra copies. An example of the former is cluster HORVU.MOREX.r3.3HG0311160, which corresponds to barley locus HvOS2 and encodes MADS-box protein ODDSOC2 [21]. This case is illustrated in Fig. 4 and Additional file 1: Fig. S12.

### Pangenes with multiple tandem copies in barley

Some gene families have tandem copies scattered in the genome. The way our protocol addresses these cases was already illustrated on Fig. 1D, with a parameter controlling how many neighbor genes of the same species can go into the same cluster. In this section, we look at how this affects the pangene clusters produced by the protocol in the same set of barley annotations used in the previous section. As we had observed that often a gene might be split in two in some assemblies, we looked for cases where specifically 3 or more gene models from the same cultivar/annotation clustered together. We found 830 such pangenes clusters out of 181,519 (0.4%). These are highly polymorphic clusters, as in 613 out of 830 (73.8%), the gene in question is missing in one or more barley cultivars. A representative example is provided in Additional file 1: Fig. S13, where a gene family is shown that has up to 6 tandem copies in some genotypes (Barke) but is missing in others (GoldenPromise). The figure also suggests these genomic regions are specially hard to annotate, as independent annotations for the same genotypes (Morex, Barke) can be quite different.

## Discussion

The protocol presented here defines pangenes across annotated assemblies of the same or related species, which in our benchmark are plant genome sets with ANI values ≥ 95. Although minimap2 worked reasonably well with the ACK2 dataset (84.7% identity), the comparison of protein sequences should perform better as the distance among the assemblies of interest grows. Indeed, whole genome alignments degrade with decreasing % sequence identity, as seen with GSAlign on the ACK2 dataset or with both algorithms when computing gene model overlap ratios. Our benchmark also considered the comparison of WGA-based clusters to more conventional approaches based on protein alignments and collinearity. This was performed on two datasets for which Compara orthogroups filtered by collinearity and gene order conservation could be retrieved. We observed that minimap2-based nucleotide clusters for ACK2 are comparable in quality to Compara orthogroups and that both minimap2 and GSAlign yield more core clusters with conserved protein domains than Compara in the rice3 dataset. In both cases, our protocol yielded more BUSCO complete core sets. We conclude that pangene clusters can be successfully derived from WGAs for closely related assemblies, which is the scope of application of our protocol. For assemblies with ANI < 80, protein-based clusters should probably be used instead.

Two algorithms for the computation of WGA were tested in this work, as we wanted to see how much the choice of aligner affected the results. This way, we also demonstrated that any aligner able to produce WGAs in PAF or other compatible formats can be integrated in this protocol. In our hands, minimap2 produced better results than GSAlign, yielding longer and more contiguous collinear regions, and pangene sets with higher BUSCO scores. For these reasons, we made minimap2 the default. However, GSAlign is superior to minimap2 in two aspects. First, it can estimate ANI values from WGAs, which measure the distances among the genomes analyzed. Second, it can cope with large WGAs such as those in the chr1wheat and barley20 datasets, provided that enough RAM is available. On the contrary, in our tests, minimap2 could not complete those alignments unless geneless regions larger than 1 Mbp were masked. We suspect this is not simply a genome size matter, as other large genomes can be aligned with minimap2, such as the human one. Instead, this is due to the repetitive nature of these genomic segments. More work is needed to explore this.

Barley pangene clusters were automatically evaluated by lifting-over CDS sequences across cultivars. This is a unique feature of the protocol presented here, as collinear, geneless genome segments can be extracted from WGAs. The results revealed that the combination of multiple annotations of the same species can potentially add a large number of intact gene models that were missing in the original annotation source. As discussed in the literature, these might be genes with low-expression or defective promoters [6, 11]. They could also be *bona fide* gene models that simply were not captured in RNAseq experiments for being expressed in specific tissues. Either way, these examples highlight one of the most important features of our protocol, that of confirming gene PAV across cultivar. Alternative approaches that do not use genome sequences, or use only gene order information, cannot carry out this task. Approaches based on protein sequences could potentially do something similar with tools such as miniprot [22].

Manual curation of a set of barley pangenes involved in flowering control was useful to further benchmark our protocol. Initially, we expected these genes to be mostly part of the core pangenome, as they all have important functions. However, we found some genes to be missing in some cultivars. In one example, we could confirm that PAV was caused by loss-of-function alleles described in the literature. In other cases, gene models could be recovered by lift-over. In another case, one cultivar had a missing gene caused by an optional algorithmic choice (-s) that restricts the computation of WGAs to pairs of homologous chromosomes and leaves unplaced contigs out of the picture. Finally, analysis of another gene revealed that calling pangenes by genomic overlap is able to group together wild type and mutated frameshifted protein-coding allelic isoforms. Together with the observed split gene models, these cases highlight the challenges of consistently annotating individuals of the same species. In fact, they confirm that analyzing soft-core, instead of core genes, is probably a good idea to tolerate the situations encountered.

The inspection of barley pangenes also helped us understand how the protocol handles gene families with tandem copies. By using default settings, we found a relatively small number of clusters with 3 or more neighbor gene models from the same cultivar. The correct handling of gene families of interest will require optimal values of the parameter (-N) that controls the maximum distance among neighbor genes to be in the same cluster. If a given family has a number of tandem copies larger than the parameter value, some copies will be placed in individual clusters.

## Conclusions

This paper presents a general protocol for the definition of pangenes in, ideally, sets of genomes of the same species. Benchmarks with several plant datasets showed that pangene clusters can be successfully derived from WGAs. Although minimap2 performed generally better than GSAlign, this required masking long repetitive parts of the genome in barley and wheat. Comparison to Compara protein-based orthogroups in rice demonstrated that pangene clusters are of similar quality in terms of encoded protein domains, while recovering more complete core sets. Further evaluation on a set of curated barley genes revealed that pangenes can successfully capture their allele diversity and helped diagnose commonly occurring situations, such as missing, inverted or split genes.

As pangenes are computed based on gene models overlapping in aligned genomes, the resulting cluster files contain all known isoforms, both as cDNA and CDS, as opposed to one sequence per gene. In this way, pangenes capture the whole annotation diversity of a gene, including potentially non-coding isoforms or frame-shifted alleles, that produce mutated protein sequences. The main advantage of building pangene clusters as described here is that genome alignments support lifting over gene models across assemblies, which can be effectively used to confirm or reject the presence-absence of certain genes. With curation work, this should improve the quality of pangene sets and, ultimately, pangenome analyses.

## Methods

### Genome sequences and genesets

A total of four datasets were used in this study (Arabidopsis ACK2, rice3, chr1wheat10 and barley20) of increasing size and complexity. They are listed in Table 1, where ACK stands for the Ancestral Crucifer Karyotype [23] and chr1wheat10 for chromosome 1 in ten different hexaploid wheats. Note that ACK2 and rice3 include different species (*Arabidopsis lyrata* and *Oryza nivara*); the others include cultivars of the same species. The ranges of average nucleotide identity (ANI) among genomes in each dataset are indicated in Table 3.

### Protocol for calling pangene clusters based on WGA evidence

The repository https://github.com/Ensembl/plant-scripts/tree/master/pangenes contains documentation, examples, and source code for calling pangenes. The main script (*get_pangenes.pl*), illustrated in Fig. 1, sequentially runs the scripts *_cut_sequences.pl*, *_collinear_genes.pl*, and *_cluster_analysis.pl*. These tasks can be performed serially on a Linux computer (default) but can also run in batches over a high performance computer cluster. Four types of sequences (cDNA, CDS [amino and nucleotide] and gDNA) are cut so that they can be subsequently added to pangene clusters. cDNA and CDS sequences are cut with GffRead with arguments -w, -y, and -x [24]. Genomic segments (gDNA) are cut with *bedtools getfasta* [25]. WGAs in PAF format are computed with minimap2 with parameters –cs -x asm20 –secondary = no -r1k,5 k [26] or GSAlign with parameters -sen -no_vcf -fmt 1 [27]. Unlike minimap2, GSAlign provides ANI estimates. Feature overlap is computed with *bedtools intersect* with parameters -f 0.5 -F 0.5 -e [25] after converting WGAs to BED files, which requires parsing the CIGAR strings contained in PAF files. When features are actual gene models, strandedness is also required. With the exception of dataset ACK2, which includes two genomes with low ANI, the analyses presented in Tables 2 and 3 were obtained with *get_pangenes.pl* and optional argument -s, which computes whole genome alignments only with homologous chromosomes. This was indicated with the regular expressions '^\d + ', '^\d + [ABD]$', and '^chr\d + H' for rice, wheat, and barley respectively.

### Pangenome terminology

We will often use pangenome-related terms to describe the pangene clusters output by our protocol. We define occupancy as the number of genomes represented in a cluster. Core clusters contain sequences from all analyzed genomes. Soft-core clusters contain sequences from 95% of the input genomes. Finally, in this paper, shell clusters are those with less occupancy than soft-core clusters after excluding singletons (occupancy = 1).

### Dotplots

The *_dotplot.pl* script can be used to make a genome-wide dotplot of collinear gene models resulting from a pairwise WGA stored in TSV format. This is done in two steps: (i) the TSV file is converted to a PAF file and (ii) the dotplot is produced with R package *pafr*, available at https://github.com/dwinter/pafr.

### BUSCO analysis

In order to evaluate the completeness of the core and soft-core collections of pangenes produced by *get_pangenes.pl*, the corresponding protein FASTA files containing all known isoforms of genes were analyzed with the conda version of BUSCO v5.4.3 [14]. The poales_odb10 lineage was selected for all datasets except ACK2, where brassicales_odb10 was used instead.

### Venn diagrams

Comparisons of CDS pangene sets produced with both WGA algorithms were carried out with script *compare_clusters.pl* from the GET_HOMOLOGUES-EST software [6]. As explained in the GitHub documentation, other scripts from this package can be used to simulate and plot the pangene set growth. The resulting Venn diagrams were plotted with Venn-Diagram-Plotter v1.6.7458 (https://github.com/PNNL-Comp-Mass-Spec/Venn-Diagram-Plotter).

### Ensembl orthologues and InterPro annotations

High-confidence orthogroups produced with Ensembl Compara [28] were retrieved with script *ens_syntelogs.pl* [29] from Ensembl Plants [30]. The following commands were used: *ens_syntelogs.pl -d Plants -c oryza_sativa -r oryza_sativa -o oryza_nivara -a* and *ens_syntelogs.pl -d Plants -c arabidopsis -r arabidopsis_thaliana -a*. These orthogroups are derived from phylogenetic trees of aligned protein sequences from most plant species in Ensembl and have extra supporting collinearity evidence. Only pairs of orthologues with WGA score ≥ 50% and gene order conservation (GOC) ≥ 75% were taken. In other words, only genes with ≥ 50% exonic coverage in whole genome alignments and 3 out of 4 conserved neighbor genes were retrieved and considered collinear (GOC allows for inversions and gene insertions). Ensembl Compara is periodically benchmarked and compared to other popular tools for orthology inference [31].

For ACK2 and rice3 genomes, pre-computed InterPro protein domains were retrieved from Biomart in Ensembl Plants [32]. These were then used to annotate the pangenes produced in this work. To check whether a pangene cluster is functionally consistent, first InterPro domains are assigned to genes in the clusters. Only if one or more domains are encoded by all genes in the cluster is it called consistent.

### Lifting-over gene models on genomic segments

The script *check_evidence.pl* uses precomputed collinearity evidence, stored in a TSV file, and lift-over alignments to project cDNA/CDS sequences on a reference genomic sequence. Briefly, collinear genome sequences are extracted with *bedtools getfasta* and then pre-clustered cDNA/CDS sequences are mapped to them with GMAP, a software tool that efficiently connects exons while accurately defining splice sites and jumping intervening introns [33]. GMAP is run with parameters -t 1 -2 -z sense_force -n 1 -F. Increasing the verbosity of the script produces the actual GMAP sequence alignments, which are useful to inspect failed lift-over attempts (i.e., partially aligned proteins, premature stop codons, length not multiple of 3).

### Flowering genes

A collection of 26 genes relevant in barley breeding due to their roles in flowering control and spike architecture was compiled. Pangene clusters were produced for the union of the barley20 dataset and two more high-quality gene annotations: MorexV3 [34] and BaRTv2 [35]. The former is the IPK annotation from http://doi.org/10.5447/ipk/2021/3 (35,826 gene models, assembly GCA_904849725.1) and the latter the JHI annotation from https://ics.hutton.ac.uk/barleyrtd/bart_v2_18.html (39,281 gene models, assembly ERS4201450). The script *get_pangenes.pl* was run with arguments -s '^chr\d + H' -H -t 0. The resulting clusters were aligned with Clustalx 2.1 for manual curation [36].

### Supplementary Information

**Additional file 1: Table S1.** Other Whole Genome Alignment stats for minimap2 and GSAlign algorithms. **Table S2.** Summary of BUSCO completeness analyses of individual genomes that are part of datasets in this paper. **Table S3.** Collinear genes found between *Arabidopsis thaliana* and *A. lyrata* within 23 blocks of the Ancestral Crucifer Karyotype based on Whole Genome Alignments produced with minimap2 and GSAlign. **Table S4.** Excerpt from BED-like pangene matrix produced during the analysis of dataset rice3. **Table S5.** Summary of Whole Genome Alignment (WGA) evidence for the gene models in CDS cluster Horvu_MOREX_1H01G011400 resulting from the analysis of dataset barley20. **Figure S1.** Overlap ratio of collinear gene models in rice, wheat and barley. **Figure S2.** Dot plots of collinear gene models called in rice, wheat and barley genomes. **Figure S3.** Venn diagrams of pangene clusters based on minimap2 and GSAlign Whole Genome Alignments of the rice3 dataset. **Figure S4.** Sequence identity among sequences in rice3 pangene clusters based on minimap2 (left) and GSAlign (right). **Figure S5.** Example of pangene cluster where the cDNA sequences have a long local alignment but the encoded CDS sequences cannot be aligned. **Figure S6.** Examples of rice pangene clusters not matched by Ensembl Compara orthogroups. **Figure S7.** Example of pangene cluster where the encoded protein sequences do not share protein domains. **Figure S8.** Flowchart of script check_evidence.pl , which uses as input a cluster in FASTA format and precomputed collinearity evidence in TSV format. **Figure S9.** Partial deletion of locus HvFT3/Ppd-H2 in barley cultivar Igri. **Figure S10.** Genomic context of pangene cluster HORVU.MOREX.r3.2HG0166090 (cluster members indicated with green arrows), which corresponds to barley locus HvCEN. **Figure S11.** Multiple alignment of protein sequences of pangene cluster HORVU.MOREX.r3.2HG0184740, which corresponds to barley locus Vrs1. **Figure S12.** Multiple alignment of protein sequences of pangene cluster HORVU.MOREX.r3.3HG0311160, which corresponds to barley locus HvOS2. **Figure S13.** Genomic context of pangene cluster gene:HORVU.MOREX.r3.7HG0752640, an example with tandem copies (cluster members indicated with green arrows), which encode acidic proteins.

**Additional file 2.** Review history.

## Data Availability

Command lines, pangene sets, and pangenome matrices of *Arabidopsis*, rice, wheat, and barley datasets are at https://github.com/Ensembl/plant-scripts/releases/download/Apr2023/pangenes_bench.tgz. FASTA & GFF files (33 GB) can be obtained from 10.5281/zenodo.7961646. The repository https://github.com/Ensembl/plant-scripts contains the documentation, examples, and source code for calling pangenes with Apache-2.0 license [37]. The version used while writing this manuscript corresponds to release Apr2023, which can be found at https://github.com/Ensembl/plant-scripts/archive/refs/tags/Apr2023.tar.gz [38].
