# Supplementary material for: GET_PANGENES: calling pangenes from plant genome alignments confirms presence-absence variation
Source: Genome Biol. 2023 Oct 5;24:223. doi: 10.1186/s13059-023-03071-z (PMC10552430; doi:10.1186/s13059-023-03071-z)
Supplement: Supplementary file 2 — Additional file 2. Review history. [file 13059_2023_3071_MOESM2_ESM.docx]

**1^st^ round**

**Reviewer 1**

The authors introduced an approach to define pangene clusters across different plant species/accessions based on whole-genome alignment. They further applied their pipeline to identify potentially mis-annotated gene models in certain genomes and also confirmed gene PAVs. The manuscript is generally clear and easy to understand. The provided scripts are useful with detailed instructions. I have some remarks:

- One of the common practices of clustering pangenes is based on amino acid sequences, such as the study in cucumber (https://www.nature.com/articles/s41467-022-28362-0). The authors applied a collinearity-based inference of gene correspondence, similar to Qin et al. used in rice (https://doi.org/10.1016/j.cell.2021.04.046) but instead of protein sequences they used genomic nucleotide sequences. A possible risk of using genomic DNA is the inter-species divergence between DNA sequences is usually higher that than of protein sequences. So it is possible that the number of DNA-based collinearity regions is underestimated. It would be helpful to add a comparison between the approach described in this study and the conventional approach based on protein synteny.
- It would be helpful to clearly indicate the advancement of the pipeline introduced in this study, compared with the existing methods to cluster pangenes that have been applied in many plant pangenome studies (https://www.sciencedirect.com/science/article/pii/S167385272200162X).
- It is not super clear why the authors used two WGA software, minimap2 and GSAlign. It turns out that minimap2 always returned better results (longer and more contiguous collinear regions, higher BUSCO completeness) and lower RAM consumption.
- A proportion of genes in plant genomes are tandemly duplicated, meaning that these genes are closely related paralogs with high sequence identity and also distributed in a vicinity. It is not stated in the current manuscript that how the pipeline handles this issue. From my personal experiences these tandemly duplicated gene-containing genomic segments may not represent very good collinearity among genomes, particularly for the NBS-LRR-encoding genes, which show high level of PAV among species/accessions.
- In page 15 the authors showed examples of PAVs from HvPPR37 and HvCEN; however, no figures or tables were linked to the results.
- The current manuscript lacks sound discussion section.

**Reviewer 2**

This article represents an important computational aid for extracting novel genes from genomes. It appears to be very competently carried out however as mentioned below some information is only provided at a rather cursory level and this needs to be expanded.

To address the questions you set.
- Are the methods appropriate to the aims of the study, are they well described, and are necessary controls included?
Yes they would seem to be. However I think that in places they could be more explicitly detailed particularly with regards to controls and benchmarks.


- Are the conclusions adequately supported by the data shown?
I feel some of the benchmarking could be more vigorously supported both statistically and within text documentation.

- Are sufficient details provided to allow replication and comparison with related analyses that may have been performed?
My feeling here is that the manuscript would benefit on a more explicit comparison here. The comparison has been done but the reader is expected to assess it themselves.

- Is the method likely to be of broad utility? Is any software component easy to install and use? Please indicate briefly the novel features and/or advantages of the method, and/or please reference the relevant publications and which methods, if any, it should be compared with.
In principle yes. the novelty is the rapid identfication of "true" pangenes. This is likely to be highly useful for a very wide range of biologists. However again I think this is not really very well self-presented by the authors. I would encourage them to better delineate what they have done and why researchers should use their method!

- Is the paper of broad interest to others in the field, or of outstanding interest to a broad audience of biologists?
I think more likely the former. However, this may be because the authors have not self-promoted well enough.

**Authors’ response**

Reviewer #1:

The authors introduced an approach to define pangene clusters across different plant species/accessions based on whole-genome alignment. They further applied their pipeline to identify potentially mis-annotated gene models in certain genomes and also confirmed gene PAVs. The manuscript is generally clear and easy to understand. The provided scripts are useful with detailed instructions. I have some remarks:

- One of the common practices of clustering pangenes is based on amino acid sequences, such as the study in cucumber (https://www.nature.com/articles/s41467- 022-28362-0). The authors applied a collinearity-based inference of gene correspondence, similar to Qin et al. used in rice

(https://doi.org/10.1016/j.cell.2021.04.046) but instead of protein sequences they used genomic nucleotide sequences. A possible risk of using genomic DNA is the inter- species divergence between DNA sequences is usually higher that than of protein sequences. So it is possible that the number of DNA-based collinearity regions is underestimated. It would be helpful to add a comparison between the approach described in this study and the conventional approach based on protein synteny.

[4] This comment touches on several issues, we will respond in parts.

Both nucleotide and protein sequences have been used in the literature to cluster pangenes. Indeed, in the mentioned cucumber study “a total of 299,692 protein-coding genes annotated from the 12 accessions were passed to GET_HOMOLOGUES-EST pipeline (v3.0.9)77 for gene clustering”, which uses nucleotide sequences, BLASTN, MCL clustering and no synteny information. As the referee mentioned, “inter-species divergence between DNA sequences is usually higher than that of protein sequences”. This increases the resolution of sequence comparisons and phylogenetic analyses, as similar genes encoding exactly the same protein can be successfully separated by comparing their codons (see for instance https://www.frontiersin.org/articles/10.3389/fmicb.2018.00771/full). This is precisely the scope of application of our protocol (see definition of pangene: a gene model or allele found in some or all individuals of a species in a similar genomic location).

However, as we have now explained in the discussion, amino acid sequence comparisons should perform better as the distance among the taxa of interest grows, as protein sequences are more conserved and support finding more remote homology relationships.

The reviewer then suggests that we compare our results with those from a “conventional approach based on protein synteny”. That’s exactly what we do in section ‘Comparison to ancestral karyotype and Ensembl orthogroups’ for two datasets which are available in Ensembl Plants (ACK2 and rice3). As explained in the Methods section, we retrieved Compara orthogroups from Ensembl Plants. These are derived from phylogenetic trees of aligned protein sequences encoded by collinear genes. Although the actual implementation is different, this approach is conceptually comparable to the gene-based pangenome produced with MCscan in the rice study cited by the referee, as both approaches protein sequences and gene order are used in the computation. Nevertheless, nucleotide and peptide-based clusters are compared in the updated Table 4, which now includes BUSCO completeness analysis. It can be seen that minimap2 nucleotide clusters for the ACK2 dataset are comparable in quality to Compara peptide clusters, and that both minimap2 and GSAlign rice3 pangenes yield substantially more complete core gene sets than Compara orthogroups, with a small drop in the functional consistency measured in terms of shared Interpro domains. These findings are now summarized in the Discussion section.

- It would be helpful to clearly indicate the advancement of the pipeline introduced in this study, compared with the existing methods to cluster pangenes that have been applied in many plant pangenome studies (https://www.sciencedirect.com/science/article/pii/S167385272200162X).

[5] Thanks for the suggestion. We have done this in the new Discussion and Conclusions sections.

- It is not super clear why the authors used two WGA software, minimap2 and GSAlign. It turns out that minimap2 always returned better results (longer and more contiguous collinear regions, higher BUSCO completeness) and lower RAM consumption.

[6] We have now added the following paragraph to the discussion:

“Two algorithms for the computation of WGA were tested in this work, as we wanted to see how much the choice of aligner affected the results. This way we also demonstrated that any aligner able to produce WGAs in PAF or other compatible formats can be integrated in this protocol. In our hands minimap2 produced better results than GSAlign, yielding longer and more contiguous collinear regions, and pangene sets with higher BUSCO scores. For this reason we made minimap2 the default algorithm of the protocol. However, GSAlign is superior to minimap2 in two aspects. First, it can estimate ANI values from WGAs, which measure the distances among the genomes analyzed. Second, it can cope with large WGAs such as those in the chr1wheat and barley20 datasets, provided that enough RAM is available. On the contrary, in our tests minimap2 could not complete those alignments unless geneless regions larger than 1Mbp were masked. We suspect this is not simply a genome size matter, as other large genomes can be aligned with minimap, such as the human one. Instead, this is most likely due to the repetitive nature of these genomic segments. More work is needed to confirm this.”

- A proportion of genes in plant genomes are tandemly duplicated, meaning that these genes are closely related paralogs with high sequence identity and also distributed in a vicinity. It is not stated in the current manuscript that how the pipeline handles this issue. From my personal experiences these tandemly duplicated gene- containing genomic segments may not represent very good collinearity among genomes, particularly for the NBS-LRR-encoding genes, which show high level of PAV among species/accessions.

[7] Thanks for this suggestion. We have now added the following section in results:

“Pangenes with multiple tandem copies in barley Some gene families have tandem copies scattered in the genome. The way our protocol addresses these cases was already illustrated on Figure 1D, with a parameter controlling how many neighbor genes of the same species can go into the same cluster. In this section we look at how this affects the pangene clusters produced by the protocol in the same set of barley annotations used in the previous section. As we had observed that often a gene might be split in two in some assemblies, we looked for cases where specifically 3 or more gene models from the same cultivar/annotation clustered together. We found 830 such pangenes clusters out of 181519 (0.4%). These are highly polymorphic clusters, as in 613 out of 830 (73.8%) the gene in question is missing in one or more barley cultivars. A representative example is provided in Supplemental Figure S13, where a gene family is shown that has up to 6 tandem copies in some genotypes (Barke) but is missing in others (GoldenPromise). The figure also suggests these genomic regions are specially hard to annotate, as independent annotations for the same genotypes (Morex, Barke) can be quite different.

- In page 15 the authors showed examples of PAVs from HvPPR37 and HvCEN; however, no figures or tables were linked to the results.

[8] The first example selected by the referee (HvPRR37) is summarized in Table 5. We have now edited this part of results, which reads as follows:

“As it turns out, the Igri genome assembly placed this gene model (Horvu_IGRI_Un01G026500) outside of the pseudo-chromosomes; instead it is located in chrUn, and therefore it cannot be collinear to the homologous genes in other cultivars when only homologous chromosomes are compared (option -s). This error can be avoided by not using option -s, which in practice means that chromosomes are compared all against all”.

We believe the current description of the observation in the text is enough, no graphics are required.

As to the second gene (HvCEN), we have now added supplementary figure S10 to illustrate its genomic context and the inversions.

Finally, note that we added a new paragraph and supplemental figure describing pangene Vrs1:

“Among the curated genes there’s also Vrs1, a homeodomain-leucine zipper homeobox gene known to control row number in barley spikes [17]. Some alleles of this locus encode proteins with frameshifts that change the phenotype, and result in aminoacid sequences that cannot be properly aligned. Supplemental Figure S11 shows that the WGA-based strategy tested here was able to cluster together these alleles despite their low terminal protein identity”.

- The current manuscript lacks sound discussion section.

[9] We have now reformatted the manuscript and added Discussion and Conclusions .

Reviewer #2:

This article represents an important computational aid for extracting novel genes from genomes. It appears to be very competently carried out however as mentioned below some information is only provided at a rather cursory level and this needs to be expanded.

To address the questions you set.

- Are the methods appropriate to the aims of the study, are they well described, and are necessary controls included?

Yes they would seem to be. However I think that in places they could be more explicitly detailed particularly with regards to controls and benchmarks.

[10] We have improved the description in Methods section ‘Ensembl orthologues and InterPro annotations’. Note there’s also a new section ‘Availability of data and materials’ where a few links are provided to obtain the input files and results of the analyses reported in this paper.

- Are the conclusions adequately supported by the data shown?

I feel some of the benchmarking could be more vigorously supported both statistically and within text documentation.

[11] We have added new BUSCO completeness results to the Results section ‘Comparison to ancestral karyotype and Ensembl orthogroups’, new examples in ‘Curation of barley flowering genes’ and added the new heading ‘Pangenes with multiple tandem copies in barley’. All previous and new results are now interpreted in the new Discussion section.

- Are sufficient details provided to allow replication and comparison with related analyses that may have been performed?

My feeling here is that the manuscript would benefit on a more explicit comparison here. The comparison has been done but the reader is expected to assess it themselves.

[12] We have updated the Methods sections ‘Ensembl orthologues and InterPro annotations’. There’s also a new section ‘Availability of data and materials’, where a few links are provided to obtain the input files and the results of the analyses reported in this paper. These include https://github.com/Ensembl/plant- scripts/releases/tag/Apr2023 , which packs all the pangene sets described in the paper and now also includes HOWTO files with the command lines required to produce them.

- Is the method likely to be of broad utility? Is any software component easy to install and use? Please indicate briefly the novel features and/or advantages of the method, and/or please reference the relevant publications and which methods, if any, it should be compared with.

In principle yes. the novelty is the rapid identfication of "true" pangenes. This is likely to be highly useful for a very wide range of biologists. However again I think this is not really very well self-presented by the authors. I would encourage them to better delineate what they have done and why researchers should use their method!

[13] We have addressed these in the new Discussion and Conclusions sections.

**2^nd^ round**

**Reviewer 1**

Thanks for the response from the authors. I am satisfied with the manuscript now.

**Reviewer 2**

The authors have very well responded to my previous concerns. I feel the mansucript is vastly improved. I have no further suggestions for improvement.
